# The association between selenium status and global and attention-specific cognition in very old adults in the Newcastle 85+ Study: cross-sectional and longitudinal analyses

**DOI:** 10.1016/j.ajcnut.2024.09.004

**Published:** 2024-09-11

**Authors:** Giorgia Perri, John C Mathers, Carmen Martin-Ruiz, Craig Parker, Kamil Demircan, Thilo S Chillon, Lutz Schomburg, Louise Robinson, Emma J Stevenson, Oliver M Shannon, Graciela Muniz-Terrera, Falko F Sniehotta, Craig W Ritchie, Ashley Adamson, Alistair Burns, Anne Marie Minihane, Jennifer Walsh, Tom R Hill

**Affiliations:** 1 Human Nutrition and Exercise Research Centre, Centre for Healthier Lives, Population Health Sciences Institute, Faculty of Medical Sciences, Newcastle University, Newcastle upon Tyne, United Kingdom; 2 MRC-Versus Arthritis Centre for Integrated Research into Musculoskeletal Ageing (CIMA), Faculty of Medical Sciences, Newcastle University, Newcastle upon Tyne, United Kingdom; 3 BioScreening Core Facility, Campus for Ageing and Vitality, Newcastle University, Newcastle upon Tyne, United Kingdom; 4 Institute for Experimental Endocrinology, Charité-Universitätsmedizin Berlin, Berlin, Germany; 5 Centre for Clinical Brain Sciences, University of Edinburgh, Edinburgh, United Kingdom; 6 Department of Social Medicine, Ohio University Heritage College of Osteopathic Medicine, Athens, Ohio, United States; 7 Centre for Dementia Prevention, University of Edinburgh, Edinburgh, United Kingdom; 8 Faculty of Medical Sciences, Population Health Sciences Institute, Newcastle University, Newcastle upon Tyne, United Kingdom; 9 Faculty of Medical and Human Sciences, University of Manchester, Manchester, United Kingdom; 10 Department of Nutrition and Preventive Medicine, Norwich Medical School, University of East Anglia, Norwich, United Kingdom; 11 Clinical Medicine, School of Medicine and Population Health, University of Sheffield, Sheffield, United Kingdom

**Keywords:** selenium status, selenoprotein P, very old adults, cognition, NuBrain

## Abstract

**Background:**

Selenium has potential safeguarding properties against cognitive decline, because of its role in protecting DNA, proteins, and lipids in the brain from oxidative damage. However, acute and chronic overexposure to selenium can be neurotoxic.

**Objective:**

The aim of this analysis was to explore the association between selenium status [serum selenium and selenoprotein P (SELENOP) concentrations and glutathione peroxidase 3 (GPx3) activity] and cognitive function in 85-y olds living in Northeast England at baseline and ≤5 y of follow-up.

**Methods:**

Global cognitive performance was assessed in 755 participants from the Newcastle 85+ study using the standardized Mini-Mental State Examination and attention-specific cognition was assessed using composite scores derived from the Cognitive Drug Research System. Serum selenium, SELENOP, and GPx3 activity were measured at baseline by total reflection X-ray fluorescence, enzyme-linked immunosorbent assay, and coupled-enzyme reaction, respectively. Regression analyses explored linear and nonlinear associations between continuous values and tertiles of selenium status biomarkers, respectively, and cognitive function at baseline. Generalized linear mixed models explored associations between continuous values and tertiles of selenium status biomarkers, and global cognitive decline over 5 y, and attention-specific cognitive decline over 3 y.

**Results:**

Over 3 and 5 y, none of the selenium biomarkers were associated with the rate of cognitive decline. At baseline, in fully adjusted models, higher serum selenium was nonlinearly associated with global cognition (*β* = 0.05 ± 0.01, *P* = 0.387 linear, *β* = 0.04 ± 0.01, *P* = 0.002 nonlinear). SELENOP and GPx3 activity were not associated with any cognitive outcomes.

**Conclusions:**

There were no associations between selenium status and cognitive decline. However, serum selenium, but not SELENOP or GPx3 activity, was positively associated nonlinearly with global cognition at baseline. Furthermore, these associations were not evident during follow-up, potentially because of residual confounding and reverse causation.

## Introduction

Dementia affects ∼55 million people globally [1] and by 2050 it is predicted that ∼152 million people will be affected [2]. In the United Kingdom, dementia has been the leading cause of death since 2012, affecting 1 in 6 people aged >80 years [3,4]. Consequently, there is a drive for further research exploring dementia, cognitive impairment and decline. Because the brain has a high oxygen consumption and rich lipid content, excessive concentrations of reactive oxygen species (ROS) can cause oxidative damage, cellular degeneration, and, over time, cognitive impairment [5]. This accumulation of oxidative stress is common in older age, in part due to an inadequate intake of nutrients with antioxidant properties, such as those found in selenium-rich food sources [6,7]. Selenium is an essential component of selenocysteine that is found in the active site of several selenoproteins including glutathione peroxidase (GPx) [[8], [9], [10]]. GPx proteins, such as GPx3, are a family of antioxidant enzymes that break down peroxides and other ROS. Observational studies have found positive associations between GPx3 activity and Alzheimer disease (AD, the main form of dementia) likely due to higher concentrations of oxidative stress that can contribute to amyloid aggregation in extracellular space [11,12] leading to GPx3 upregulation [9,[13], [14], [15], [16], [17]]. Selenoprotein P (SELENOP) is a secreted glycoprotein, produced mainly by the liver. SELENOP acts as a transporter to deliver selenium to the brain by binding to the surface receptor apolipoprotein E receptor 2 (ApoER2) [18,19] and prioritizes selenium delivery to the brain during selenium deficiency [20]. Several selenoproteins, including SELENOP, are essential for brain development and genetic knockout of these proteins causes embryonic lethality in mice [21,22]. Studies in mice [18,22,23], as well as cross-sectional [[24], [25], [26], [27], [28], [29]] studies in humans, provide evidence that low SELENOP concentrations are associated with poorer cognitive function. Lower serum SELENOP concentration (≤2.3 mg/L) in patients hospitalized for heart failure was associated with higher odds of cognitive impairment. Likewise, various observational studies have also found positive associations between serum selenium and cognitive performance [[30], [31], [32], [33], [34]]. Although other studies have reported negative or null relationships [35,36] and some reviews and mechanistic studies have revealed nonlinear relationships between selenium status and cognitive function [18,37,38], a 2-sample Mendelian randomization study found that individuals with higher genetically determined selenium may have a higher risk of developing AD [39]. Furthermore, serum selenium concentrations may not reflect selenium concentrations in the brain and cerebral spinal fluid (CSF) for example, higher selenium concentrations were found in CSF of cases with neurodegenerative disease [40,41]. Excessive (inorganic) selenium exposure has been cautioned as a potential risk factor for neurodegenerative and neuropsychiatric disease [42] and an observational study in those with mild-cognitive impairment found serum and CSF SELENOP concentrations to be nonlinearly associated with dementia risk [43]. Nevertheless, low selenium status has been associated with higher concentrations of inflammatory markers, such as IL-6 and TNF-α [44,45] that play a fundamental role in chronic disease development, including cognitive decline and dementia pathogenesis [38,46,47]. Despite selenium’s importance in health, deficiency is common in older adults. In a United Kingdom-based cohort study (Newcastle 85+ study), mean selenium intake (*n =* 781) was below the lower reference nutrient intake [48] and furthermore, 82% of the cohort had suboptimal selenium status (≤70 μg/L) [49]. Currently, there is a lack of information on the association between multiple selenium biomarkers and cognitive decline in very old adults (≥85 y), among whom >18% are estimated to have dementia [50,51]. This is crucially important given that the proportion of very old adults in the United Kingdom is predicted to more than double between 2019 and 2040 [52]. This analysis aimed to investigate the associations between selenium status (measured using serum selenium and SELENOP concentrations and GPx3 activity) and cognitive function in very old adults from the Newcastle 85+ Study, both cross-sectionally and prospectively, for ≤5 y. It was hypothesized that higher selenium status would be associated with better cognitive function and lower selenium status would be associated with greater rate of cognitive decline during follow-up.

## Methods

### Participants

The Newcastle 85+ Study is a population-based, longitudinal study of health trajectories and outcomes in very old adults who were born in 1921. Recruitment included those permanently registered with a participating general practice in Newcastle upon Tyne or North Tyneside (Northeast of England) primary care trusts. The only exclusion criteria were those with end-stage terminal illnesses and those who may pose a safety risk to a lone nurse visiting the participant. The sample size for the initial study in 2006 was determined by increasing the sample size by 1/3rd of a previous, similar study in very old adults (Leiden 85+ Study), as well as a pilot study and statistical calculations [53]. At the time of recruitment (June 2006–November 2007 [53]), the study cohort was sociodemographically representative of the general United Kingdom population and included institutionalized older adults. A flowchart of participants used in this study can be found in the Supplementary Material (Supplemental Figure 1). Full study details can be found in previous publications [54] and study questionnaires can be found at http://research.ncl.ac.uk/85plus.

### Ethical approval

The study was conducted in accordance with the Declaration of Helsinki and the Newcastle and North Tyneside local research ethics committee (06/Q0905/2) approved the research. Written informed consent was obtained from all participants, or from a caregiver or relative according to the UK Mental Capacity Act 2005.

### Cognitive assessments

The standardized Mini-Mental State Examination (SMMSE) was used to assess global cognitive status at baseline, 1.5-, 3-, and 5-y follow-up. The SMMSE is a brief dementia screening instrument that provides a global score of cognitive function ranging from 0 to 30 points and that correlates well with activities of daily living [55]. The Cognitive Drug Research (CDR) computerized assessment system was used to measure attention at baseline, 1.5- and 3-y follow-up. A high-resolution Windows-based laptop computer (Motion Computing LE1600 Tablet PC with keyboard accessory) was used to display the CDR tasks that took ∼15 min to complete. Responses were recorded using a 2-button (YES/NO) response box. Before the study measurements, a familiarization session (with fewer stimuli) was undertaken ∼1 wk earlier to ensure participants understood the testing procedures. Trained nurses provided standardized, verbal instructions where each participant had access to the same research nurse. In cases where instructions were misunderstood, the instructions were repeated/reworded. Similarly, tasks could be paused, restarted, or repeated due to misunderstanding only. If a participant was agitated by the tasks, for example, by being distressed by their performance or unable to understand the task despite repeated explanations, the task could be omitted or aborted at the discretion of the research nurse. Each session was recorded electronically to report task completion and reasons for missing data, if any. Further details on the scores and validation can be found in the Supplementary Material and in a previous publication [56]. Attention tasks comprised of mean reaction times (speed scores) of correct responses (in milliseconds). The tests carried out were simple reaction time (SRT), which measures alertness and concentration; choice reaction time (CRT), which also measures alertness and concentration with an additional section on information processing speed; and digit vigilance task (DVT), which measures sustained attention and the ability to ignore distractions [56]. Three validated composite measures were derived from these tasks [57,58]: power of attention (PoA), a sum of 3 attention speed scores (SRT, CRT, and DVT mean reaction times in ms) that measures the intensity of concentration and the ability to focus attention; continuity of attention (CoA), which assesses the ability to sustain attention during the testing period and combines the accuracy scores from CRT and DVT (CRT accurate responses × 0.30 + DVT accurate responses × 0.30 – DVT false alarms); and reaction time variability (RTV), which is the sum of the coefficients of variance (CV) of the reaction (speed) time scores (SRT, CRT, and DVT mean reaction times) and reflects fluctuation in attention and consistency in responding to correct stimuli. Tolerances on display onset times and the measurements of RTV are unknown. For SMMSE and CoA, higher scores indicate better function, whereas lower scores for PoA and RTV indicate better function.

### Biomarkers of selenium status

Baseline serum samples were collected in 2006–2007 for 757 participants and were stored at −80°C. Samples were analyzed for biomarkers of selenium status: selenium (μg/L) and SELENOP (mg/L) concentrations and GPx3 activity (U/L). Methodological details can be found elsewhere [49]. In brief, total serum selenium concentration was measured by total reflection X-ray fluorescence (TXRF) using a bench-top spectrometer (S4 T-STAR, Bruker Nano GmbH). Serum standard Seronorm was used as a control (concentration of 87 μg/L determined by inductively coupled plasma mass spectrometry) (Seronorm Trace Elements Serum L-1 Seronorm, Cat#201405, Lot-Nr 1309438, Sero AS). The inter- and intra-assay CVs from the 10 assay runs were <10% at 76–99 μg/L. The lower detection limits for selenium range from 0.32 to 0.49 μg/L [59]. Serum SELENOP concentration was analyzed using a validated immunoluminometric, commercial ELISA (selenOtest, selenOmed GmbH). A photometer was used to measure absorbance at 450 nm. Each sample was measured in duplicate, and the mean SELENOP concentrations were calculated. GPx3 activity was analyzed using a coupled-enzyme reaction measuring nicotinamide adenine dinucleotide phosphate hydrogen (NADPH) consumption [60]. Serum samples (including control serum) were incubated at 20°C with 0.27 mg/mL NADPH, 1 mM sodium azide, an enzyme buffer containing 3.4 mM reduced glutathione and 0.3 U/mL glutathione reductase. The reaction was initiated using hydrogen peroxide. At 340 nm, reductions in UV absorption were proportional to NADPH consumption, which reflected GPx3 activity.

### Assessment of other covariates

At baseline, participants consented to multidimensional health assessments comprising questionnaires, blood-related measurements, dietary intake, and functional tests [53,61]. General practitioner records were reviewed to extract data on diagnosed diseases [diabetes, cardiovascular and neurocognitive (Parkinson and dementia) conditions] and prescribed medication. Assessments were conducted in each participant’s place of residence by a trained research nurse. Physical activity was categorized as low/moderate/high (score 0–1/score 2–6/score 7–18, respectively) using a validated purpose-built questionnaire [62]. Selenium intake was determined using the 24-h multiple pass recall [63]. Smoking status and alcohol intake, assessed by nurses during participant interviews, were categorized as current/former/never [64] and depression was assessed using the 15-item Geriatric Depression Scale (GDS). The covariates used in these analyses were informed from previous research on nutritional status and cognitive impairment in the Newcastle 85+ Study [56,65].

### Statistical analyses

Statistical analyses were conducted using the IBM statistical tool SPSS (v27.0) and R (v4.3.2) with packages “ggplot2,” “Hmisc,” and “dplyr” for exploring nonlinear associations at baseline and at each time point during follow-up. Normality was assessed with the Shapiro–Wilk test and confirmed with Q–Q plots and *P* < 0.05 was considered statistically significant. Normally distributed continuous data are presented as mean values and SDs, whereas non-normally distributed data are presented as median and interquartile ranges. Descriptive statistics were used to summarize the baseline characteristics of all participants and of those with biomarker concentrations in each tertile. Differences in characteristics between tertiles were assessed using Chi-square test (categorical) and Kruskal–Wallis (for ordinal and non-normally distributed data).

Previous analyses in this cohort investigating selenium status and its determinants utilized a binary cut-off based on biologically derived thresholds, that is, potential optimal selenium status [49]. However, because of the prevalent suboptimal selenium status in this population and the potential for nonlinear relationships with cognitive decline, in this analysis we used both continuous measures (main text) and tertiles (supplementary) of selenium status biomarkers. Data distributions for SMMSE and attention-specific outcomes were tested for normality and transformations (natural log and box-cox) were applied, although this did not result in normalization of the residuals in all models. However, the residuals from the models of RTV, PoA, and CoA were approximately normal and did not interfere with convergence.

Linear regression models were fitted to assess the cross-sectional associations between selenium status and mean global cognition scores (SMMSE) and attention-specific scores (CDR). R was also used to fit cubic splines on regression models with 3 knots at the 10th, 50th, and 90th percentiles for associations between the biomarkers of selenium status and cognitive outcomes at baseline. Nonlinearity was determined using likelihood ratio test. For the longitudinal analyses, generalized linear mixed models were fitted due to their tolerance of non-normally distributed residuals. These were used to assess the change in SMMSE between baseline and 1.5-, 3-, and 5-y of follow-up and separate models for each attention-specific score (PoA, CoA, and RTV) between baseline and 1.5 and 3 y. To facilitate comparison and improve model performance, continuous predictor variables (time, serum selenium, SELENOP, GPx3 activity, selenium intake, waist:hip ratio, GDS score, and disease count) were standardized and verified by checking the means (∼0) and standard deviations (∼1). Using the “glmer” function from the “lme4” package, generalized linear mixed models with a Gamma distribution (for skewed distributions of outcome variables), log link function, and “bobyqa” optimizer were employed alongside random intercepts to account for individual differences. Time in years was used as a time-varying covariate and baseline selenium status in tertiles (serum selenium, SELENOP, and GPx3 activity) were used as independent variables to predict the rate of change in the outcome measures. Covariates included in all models (both cross-sectional and longitudinal) were as follows: Model 1 included the relevant selenium status biomarker and time interaction (in longitudinal analyses). Model 2 was adjusted for variables in Model 1 plus GDS, diabetes, cardiovascular and neurocognitive conditions, sex, education, alcohol consumption, physical activity, smoking status, waist:hip ratio, and disease count. A fixed quadratic slope was also tested to determine the nonlinearity of the models and Bayesian information criterion (BIC) indices of the linear and quadratic models were compared. With the exception of the SMMSE models that were better suited to quadratic models, linear models were selected to provide a more parsimonious model and to prevent overfitting. To visualize the nonlinear relationship between the selenium biomarkers and cognitive outcomes, regression plots with restricted cubic splines (RCSs) were used to model potential nonlinear relationships at each time point.

In the sensitivity analyses, the models were reanalyzed using the tertiles of selenium biomarkers as well as separate models excluding those with neurodegenerative disease and including those living in institutions (Supplementary Material).

## Results

### Population characteristics by biomarkers of selenium status

Table 1 shows the baseline characteristics, for the entire cohort (*n =* 755, mean age: 85.4 ± 0.4 y, BMI: 24.4 ± 4.3 kg/m^2^) and by tertiles of serum selenium concentration (<46.7, 46.3–62.0, and ≥62.0 μg/L). Those with serum selenium concentrations in the highest tertile compared with the lowest were more likely to have higher scores for SMMSE and CoA at baseline (Table 2). There were no significant differences in cognitive measures between tertiles of SELENOP concentration or GPx3 activity at baseline (Supplemental Table 1). At 1.5 y (*n =* 598), those with serum selenium concentrations in the highest tertile were more likely to have higher scores for SMMSE and CoA and lower scores for RTV. Similarly, at 3 y (*n =* 472), those with serum selenium concentrations in highest tertile were more likely to have higher scores for SMMSE and CoA and at 5 y (*n =* 354) they had higher scores for SMMSE (Table 2). For details on attrition see Supplementary Table 2 [49]).

**TABLE 1:** Baseline characteristics by serum selenium tertiles.

Characteristic	All participants	Serum selenium (μg/L)		
		<46.7	46.3–62.0	≥62.0
**Participants (*n*)**	755	253	251	251
**Females, % (*n*)**	61.1 (461)	60.5 (153)	57.8 (145)	64.9 (163)
**Dietary selenium intake (μg/d)**	39.1, 29.2	35.8, 24.1	39.8, 29.8	41.9, 31.9
**Serum selenium (μg/L)**	53.6, 23.6	38.02, 12.3	53.7, 7.7	70.8, 12.6
**SELENOP (mg/L)**	2.9, 1.9	2.2, 1.2	3.1, 1.5	4.0, 2.4
**GPx3 activity (U/L) Mean, SD**	144.0 ± 50.7	121.08 ± 2.77	146.47 ± 2.91	164.77 ± 3.28
**Waist:hip ratio**	0.9, 0.1	0.9, 0.1	0.9, 0.1	0.9, 0.1
**Education**				
**0–9 y**	63.2 (477)	65.6 (162)	24.3 (60)	10.1 (25)
**10–11 y**	23.2 (175)	66.4 (164)	20.6 (51)	13.0 (32)
**12+ y**	12.1 (91)	60.6 (151)	25.7 (64)	13.7 (34)
**Occupation**				
**Managerial and professional**	33.5 (253)	34.2 (81)	11.4 (27)	54.4 (129)
**Intermediate**	14.0 (106)	33.8 (81)	14.6 (35)	51.7 (124)
**Routine and manual**	47.9 (362)	37.3 (91)	18.0 (44)	44.7 (109)
**Physical activity**				
**Low**	21.5 (162)	32.1 (80)	16.9 (42)	16.0 (40)
**Medium**	42.6 (322)	38.6 (96)	49.0 (122)	41.6 (104)
**High**	35.0 (264)	29.3 (73)	41.6 (104)	42.4 (106)
**Number of medications**	6.0, 5.0	7.0, 5.0	6.0, 5.0	5.0, 5.0
**Geriatric depression scale**				
**None**	73.9 (558)	75.0 (168)	81.0 (192)	81.8 (198)
**Mild**	11.7 (88)	17.0 (38)	9.7 (23)	11.2 (27)
**Severe**	7.5 (57)	8.0 (18)	9.3 (22)	7.0 (17)
**Institutionalized, % (*n*)**	8.9 (67)	18.2 (46)	5.2 (13)	3.2 (8)
**Alcohol drinkers, % (*n*)**	60.7 (458)	53.4 (134)	66.0 (165)	63.6 (159)
**Smokers, % (*n*)**	5.6 (42)	6.3 (16)	3.6 (9)	6.8 (17)
**Cardiovascular conditions, % (*n*)**	78.3 (591)	80.6 (203)	81.7 (205)	72.9 (183)
**Diabetes, % (*n*)**	14.2 (107)	33.6 (36)	16.8 (42)	11.6 (29)
**Dementia/Alzheimer/Parkinson, % (*n*)**	9.1 (69)	13.5 (34)	8.0 (20)	6.0 (15)

Abbreviations: GPx3, glutathione peroxidase 3; hsCRP, high-sensitivity C-reactive protein; SELENOP, selenoprotein P.

Participants were compared between low, medium, and high serum selenium concentrations using chi-square test for nominal values and Kruskal–Wallis for ordered and non-normally distributed data. All values represent median and IQR, unless otherwise stated.

**TABLE 2:** Measures of cognitive function at baseline and at 1.5-, 3-, and 5-y follow-up according to tertiles of serum selenium concentration at baseline.

Characteristic	All participants	Serum selenium (μg/L)			*P*
		<46.7	46.3–62.0	≥62.0	
**Baseline**					
**SMMSE**	26.1 ± 4.9	24.9 ± 0.37	26.3 ± 0.32	27.1 ± 0.22	<0.001
**PoA (ms)**	1503.4 ± 210.4	1520.5 ± 207.2	1505.7 ± 218.7	1486.2 ± 204.6	0.186
**CoA (ms)**	51.8 ± 8.7	50.1 ± 0.7	52.0 ± 0.5	53.2 ± 0.4	0.005
**RTV (ms)**	64.1 ± 19.5	67.4 ± 1.6	63.2 ± 1.1	62.0 ± 1.1	0.123
**1.5 y**					
**SMMSE (*n*)**	26.7 ± 0.2	25.8 ± 0.4	26.6 ± 0.3	27.4 ± 0.2	0.006
**PoA (ms)**	1599.7 ± 326.0	1648.7 ± 367.5	1609.4 ± 325.7	1554.4 ± 286.8	0.058
**CoA (ms)**	51.8 ± 0.4	51.3 ± 0.7	51.8 ± 0.6	52.3 ± 0.6	0.027
**RTV (ms)**	64.0 ± 0.9	67.7 ± 1.9	63.9 ± 1.4	61.4 ± 1.5	0.002
**3 y**					
**SMMSE (*n*)**	25.5 ± 0.3	24.5 ± 0.6	25.8 ± 0.4	26.1 ± 0.4	0.042
**PoA (ms)**	1631.2 ± 391.3	1636.9 ± 413.3	1657.6 ± 413.1	1603.9 ± 355.5	0.383
**CoA (ms)**	51.9 ± 0.4	51.4 ± 0.8	51.3 ± 0.7	52.7 ± 0.7	0.012
**RTV (ms)**	63.1 ± 1.1	63.8 ± 2.3	63.9 ± 1.7	61.9 ± 1.7	0.476
**5 y**					
**SMMSE (*n*)**	24.9 ± 0.4	23.3 ± 0.9	24.5 ± 0.6	26.3 ± 0.5	0.001

Abbreviations: CoA, continuity of attention; PoA, power of attention; RTV, reaction time variability, SMMSE, standardized Mini-Mental State Examination.

### Selenium status and cognitive function

**FIGURE 1:**
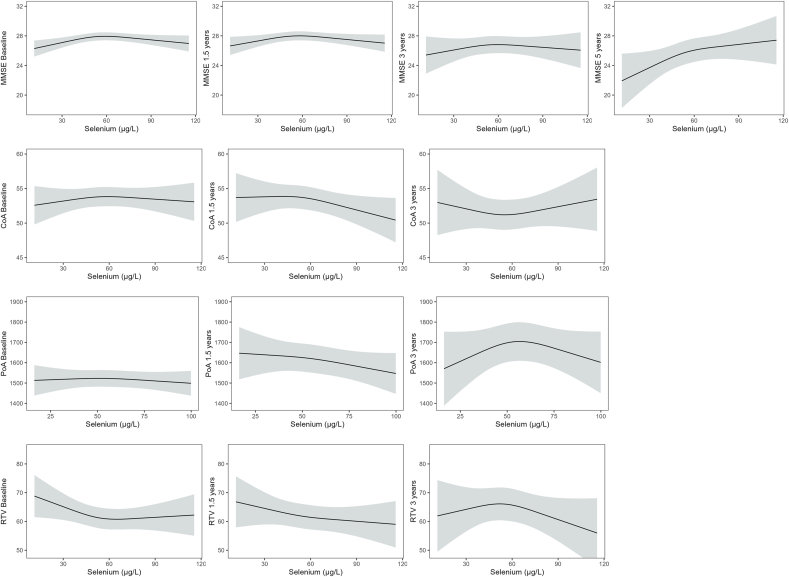
Restricted cubic splines for serum selenium and standardized Mini-Mental State Examination (SMMSE), continuity of attention (CoA), power of attention (PoA), and reaction time variability (RTV), using a priori knots at 5th, 10th, and 90th percentiles. Estimates from the fully adjusted models are depicted by solid lines and 95% confidence intervals are depicted by the shaded areas. Models are adjusted for biomarker of interest, sex, physical activity, waist:hip ratio, education, geriatric depression score (GDS), disease count, presence of all diabetes, cardiovascular and neurocognitive conditions, smoking status, and alcohol intake and selenium intake.

At baseline, none of the biomarkers of selenium status were associated with any cognitive outcome in the fully adjusted models (Table 3). In contrast, participants in the highest tertile of serum selenium concentration had SMMSE that was on average 0.58 points (SE = 0.27, *P =* 0.032) greater than those in the lowest tertile after adjustment for all covariates (Supplemental Table 3). The linear and nonlinear relationships between biomarkers of selenium status and cognitive outcomes at baseline are presented in Figure 1 and the comparison of linear and nonlinear models in Table 4. The latter shows that there was evidence of a nonlinear relationship between serum selenium concentration and SMMSE that increased with increasing serum selenium until reaching an inflexion point/plateau at ∼55–60 μg/L serum selenium. Inspection of these RCS plots (Figure 1) suggests that there may be a similar inflection point between serum selenium concentration and other measures of cognitive function at each time point during follow-up. However, we found little evidence for links between other markers of selenium status (SELENOP concentration and GPx3 activity) and any measure of cognitive function at any time point (Supplemental Figures 2 and 3).

**TABLE 3:** Relationships between measures of cognition and tertiles of each biomarker of selenium status (serum selenium, glutathione peroxidase 3 activity, selenoprotein P).

Outcome	Serum selenium		Selenoprotein P		GPx3 activity	
	*β* (SE)	*P*	*β* (SE)	*P*	*β* (SE)	*P*
**SMMSE Model 1**	0.05 (0.01)	<0.001	0.01 (0.13)	0.519	2.75^E-3^ (3.58^E-3^)	0.442
**Model 2**	0.05 (0.01)	0.387	0.03 (0.08)	0.741	−1.53^E-3^ (2.15^E-3^)	0.477
**PoA Model 1**	−0.94 (0.44)	0.033	4.17 (5.86)	0.480	0.27 (0.16)	0.100
**Model 2**	−0.25 (0.45)	0.580	5.53 (5.73)	0.335	0.23 (0.16)	0.142
**CoA Model 1**	0.05 (0.02)	0.001	0.15 (0.22)	0.474	0.01 (0.01)	0.244
**Model 2**	3.77^E-3^ (0.02)	0.806	−0.15 (0.20)	0.439	2.25^E-4^ (5.49^E-3^)	0.967
**RTV Model 1**	−0.10 (0.04)	0.010	−0.83 (0.51)	0.106	−0.01 (0.01)	0.304
**Model 2**	−0.06 (0.04)	0.151	−0.75 (0.51)	0.142	−3.69^E-3^ (0.01)	0.796

Abbreviations: CoA, continuity of attention; GPx3, glutathione peroxidase activity; PoA, power of attention; RTV, reaction time variability; SMMSE, standardized Mini-Mental State Examination.

Model 1: adjusted for biomarker of interest; Model 2: adjusted for biomarker of interest, sex, physical activity, waist:hip ratio; education, geriatric depression score (GDS), disease count, presence of all diabetes, cardiovascular and neurocognitive conditions, smoking status, alcohol, and selenium intake. The lowest selenium biomarker was set as the comparator. Lower *β* scores for PoA and RTV and higher *β* scores for CoA and SMMSE indicate better function. SeTert SePP = 621 GPx3 = 619 PoA, RTV; CoA 623, 621, 639, 637 SMMSE.

**TABLE 4:** Likelihood ratio tests between linear and nonlinear associations between each biomarker of selenium status (serum selenium, glutathione peroxidase 3 activity, selenoprotein P) and cognitive outcomes at baseline derived using restricted cubic splines.

Outcome	Serum selenium		Selenoprotein P		GPx3 activity	
	*χ*^2^	*P*	*χ*^2^	*P*	*χ*^2^	*P*
SMMSE	9.12	<0.001	1.10^E-2^	0.973	0.07	0.793
PoA	2.93	0.087	0.18	0.675	0.41	0.521
CoA	0.48	0.489	0.01	0.923	0.74	0.391
RTV(ln)	1.20	0.274	4.00^E-4^	0.985	0.10	0.754

Abbreviations: CoA, continuity of attention; GPx3, glutathione peroxidase activity; PoA, power of attention; RTV, reaction time variability; SMMSE, standardized Mini-Mental State Examination.

Significance denotes a nonlinear association determined using *n =* 755. All models had degrees of freedom equivalent to 1.

### Selenium status and rate of cognitive decline

All the outcome measures declined (lower scores for SMMSE and CoA and higher scores for PoA and RTV) with time of follow-up in fully adjusted models; analyses using measures of selenium status as continuous variables showed no significant associations between any measure of selenium status and rate of decline in cognitive function for any of the outcome measures over ≤5 y of follow-up (Table 5). In contrast, there was evidence that those participants in the highest tertile of serum selenium concentration had a lower rate of decline in SMMSE compared with those in the lowest tertile in the fully adjusted model [*β* = 0.04 (0.02), *P =* 0.022] (Supplemental Table 4). There were no apparent associations between of rate of decline in cognitive function and biomarkers of selenium status (SELENOP and GPx3) in the fully adjusted models (Supplemental Table 4).

**TABLE 5:** Associations between tertiles of selenium status biomarkers at baseline and cognitive decline (SMMSE) over 5 y and composite scores of cognition over 3 y using generalized linear mixed models.

Outcome	Serum selenium		Selenoprotein P		GPx3 activity	
	*β* (SE)	*P*	*β* (SE)	*P*	*β* (SE)	*P*
**SMMSE Model 1^1^**	0.03 (0.01)	<0.001	0.01 (0.01)	0.207	0.01 (0.01)	0.414
**Model 2**	−0.01 (0.01)	0.236	6.05^E-3^ (0.01)	0.432	4.49^E-4^ (0.01)	0.953
**PoA Model 1^2^**	−0.02 (0.01)	0.083	2.83^E-3^ (0.01)	0.750	0.01 (0.01)	0.160
**Model 2**	−4.85^E-3^ (0.01)	0.573	4.53^E-3^ (0.01)	0.591	7.55^E-3^ (0.01)	0.358
**CoA Model 1^2^**	0.03 (5.58^E-3^)	<0.001	5.01^E-3^ (0.01)	0.670	2.80^E-3^ (5.33^E-3^)	<0.001
**Model 2**	−1.60^E-3^ (0.01)	0.866	−0.01 (0.01)	0.382	−4.35^E-3^ (0.01)	0.635
**RTV Model 1^2^**	−0.03 (0.01)	0.012	−0.01 (0.01)	0.416	−0.01 (0.01)	0.466
**Model 2**	−0.02 (0.01)	0.166	−0.01 (0.01)	0.367	−2.03^E-3^ (0.01)	0.868

Abbreviations: CoA, continuity of attention; GPx3, glutathione peroxidase activity; PoA, power of attention; RTV, reaction time variability; SMMSE, standardized Mini-Mental State Examination; Time, change over time.

^1^Decline over 5 y.

^2^Decline over 3 y. Results produced from generalized linear mixed models. Model 1: adjusted for biomarker of interest and time; Model 2: adjusted for biomarker of interest, time and their interaction, sex, physical activity, waist:hip ratio, education, geriatric depression score, disease count, presence of all diabetes, cardiovascular and neurocognitive conditions, smoking status, alcohol intake, and selenium intake. For all models, tertile 1, the lowest concentration, was used as the reference (0.00). Lower *β* scores for PoA and RTV and higher *β* scores for CoA and SMMSE indicate better function. SMMSE *N =* 753, 588, 441, 313 for baseline, 1.5-, 3-, and 5-y follow-up; CoA and PoA *N =* 705, 528, 395, and RTV *N =* 702, 528, 393 for baseline, 1.5- and 3-y follow-up.

## Discussion

### Main findings

In people initially aged 85 y, there was sparse evidence that selenium status was associated with cognitive function or with rate of decline in measures of cognitive function over ≤5 y of follow-up. However, there was some evidence of a nonlinear association between serum selenium concentration and global cognition (measured as SMMSE) with higher SMMSE in participants with serum selenium concentrations around 55–60 μg serum selenium/L than in those with lower serum selenium concentration. Furthermore, analyses using tertiles of serum selenium concentration suggested that rate of decline in SMMSE was lower for those in the highest compared with lowest tertile. In contrast, there was no evidence of associations in rate of decline in other measures of cognitive function and with other biomarkers of selenium status over 3–5 y of follow-up.

### Comparison with other studies

Contrary to our hypotheses and to reports from other previous studies [30,31,34,38,46,66,67], we did not find associations between SELENOP concentration or GPx3 activity and measures of cognitive function, or decline in measures of cognitive function, in fully adjusted models. In a longitudinal study of participants initially aged 61–70 y (*n =* 1389) in France [Etude du Vieillissement Artériel (EVA) study], plasma selenium concentration was measured at baseline and after 9 y. For participants in the EVA study, the greater the decrease in plasma selenium, the higher the probability of cognitive decline. Among Italian adults aged 65+ y (*n* = 1012), plasma selenium concentration (mean 74.5 μg/L) was positively associated with time-based coordination tasks involving rapid alternating movements (finger-tapping and pronation and supination of hands) [33]. In the NHANES, higher serum selenium (*n =* 2146, ≥60 y) was associated with better cognitive performance assessed using the Consortium to Establish a Registry for AD for immediate and delayed memory, and the Digit Symbol Substitution Test for working memory [67], with similar positive associations reported in another analysis using NHANES [66]. Likewise, a cross-sectional study (*n* = 2000) of Chinese adults aged 65+ y reported that lower selenium status (determined using toenail samples) was associated with greater cognitive decline assessed using the Community Screening Interview for Dementia and Indiana University Token Test that assess working memory and executive function [34]. However, the cognitive outcome measures used in these studies (coordination and working memory) did not test the same domains as our study (global cognition and attention). In addition, participants in these studies were considerably younger (on average) than those in our Newcastle 85+Study. However, in a relatively small cross-sectional study of older Australian adults (mean age 71 y), no association was found between selenium status and cognitive performance possibly because selenoprotein synthesis was optimized due to adequate selenium intake (93.1 μg/d) and a relatively high plasma selenium concentration (169.3 μg/L) [36]. In the Prevention of Alzheimer's Disease by Vitamin E and Selenium trial, supplementation with selenium (200 μg/d for a mean 5.4 y, L-selenomethionine) had no effect on dementia incidence after a 7-y follow-up (males *n =* 3768, ≥60 y) [68]. The nonlinear associations between serum selenium and global cognition at baseline suggests that there may be an optimal concentration of serum selenium required for cognitive function akin to the U-shaped curve phenomenon between selenium status and health and mortality [69] and to the L-shaped relationship observed between selenium status and all-cause mortality in older German adults [70].

Multiple factors, such as medication use (i.e. proton pump inhibitors and statins that can reduce selenium absorption), the presence of disease and nutrient interactions (i.e. zinc [71], iron [72], and copper [73]), can have adverse effects on selenium status. The use of multiple medications increases with age and polypharmacy was common among participants in the Newcastle 85+ Study [74] that may obscure relationships between selenium status and cognitive function among the very old. Methodological differences between studies and the heterogeneity of different populations may further explain the differences in findings or lack of associations with SELENOP and GPx3 activity. For example, based on our understanding, no other studies exploring selenium status and cognition have used TXRF to measure selenium status [35]. Similarly, to our knowledge, the CDR assessment has been used in only one study of patients with AD that found no association between selenium status and baseline cognitive function [75]. Finally, in the Newcastle 85+ Study, it is not known whether the serum selenoprotein concentrations measured in blood reflect the concentrations in the brain. Concentrations of selenium in brain regions are varied; some studies have suggested widespread deficiency in brain tissue of those with Huntington disease [76] or higher concentrations in the gray matter and lower in the cerebral cortex [77]. It may be helpful to utilize selenoprotein concentrations from CSF [24] as a surrogate for concentrations in the brain. In one such study, higher SELENOP concentrations in both serum and CSF were associated nonlinearly with dementia risk [43]. The lack of associations between the measured selenoproteins and cognitive function could suggest that other selenoproteins may play a larger role in brain health [46]. During times of selenium deficiency, the expression of certain selenoprotein genes such as *GPX1*, *GPX3*, and *selenoprotein W (SELENOW*) are more sensitive and thus downregulated compared with “housekeeping” selenoproteins such as deiodinases (*DIO*s) and *GPX4* [78]. In addition to this molecular hierarchy, there is a tissue hierarchical system where selenium is preferentially retained in certain organs during selenium deficiency, such as the brain and reproductive organs [79]. Serum selenium may act independently of selenoproteins through neurotransmitter regulation [42], insulin-like growth factor 1 signaling [80,81], or thyroid function [82,83].

### Strengths and limitations

To our knowledge, this is the first prospective study to explore linear and nonlinear associations between multiple biomarkers of selenium status and cognitive function in very old adults, at baseline and for ≤5 y of follow-up. The study included validated attention-specific cognitive measures that were pilot tested in this age group [56] alongside the validated SMMSE that is universally understood and the most common global cognitive screening tool used by clinicians [84]. The Newcastle 85+ Study was sociodemographically representative of the general United Kingdom population and included people living in institutions and those who were cognitively impaired; 2 groups commonly excluded from previous studies. Finally, the use of a single birth cohort reduced the heterogeneity that can confound other study designs involving multiple birth years.

However, as with all secondary analyses of observational data, residual confounding may have influenced the observed association between selenium status and cognition. However, this was reduced by adjusting for appropriate confounders and performing sensitivity analyses. This study did not account for APOE ε4 carrier status, although a recent study found that associations between diet and dementia risk were independent of genetic predisposition [85]. Reverse causation in the cross-sectional analyses may explain the findings such that poorer cognitive function may lead to a lower selenium intake [86] and conversely cognitive impairment may be a consequence of these dietary or status changes. Despite the SMMSE and CDR assessments offering valuable and appropriate insights into the cognitive performance of very old adults, the SMMSE may not detect subtle changes such as mild-cognitive impairment due to ceiling effects [87]. Additionally, because this is an analysis of archived data, we do not have details of the tolerances in the display onset times used for the measurements of RTVs, which may have added further variation. Furthermore, the measures of this specific cohort did not include aspects of memory or sensory processing, both important components in cognitive performance and decline. However, processing speed and attention have been proposed to decline before memory [88]. Finally, as with all analyses in very old adults, the participants who survived the study duration may have different lifestyle and genetic predispositions warranting them to live longer than participants who died during this time. Thus, although this analysis provides for the first time insights into selenium status and cognition in very old adults, the associations may not reflect all aging populations.

### Conclusions

In summary, all 3 biomarkers of selenium status were not predictive of the rate of cognitive decline. However, serum selenium, ≤55 to 60 μg/L was associated with better cognitive function in very old adults although higher concentrations do not appear to offer any further benefit and may be detrimental. Future prospective analyses could explore the selenium concentration in post-mortem brain tissue of very old adults with mild-cognitive impairment and other selenoproteins (i.e. selenoprotein S, selenoprotein M, and DIOs) to improve our understanding of the association between selenium status and cognition.

## Author contributions

The authors’ responsibilities were as follows – GP, JCM, TRH: designed research; GP, CM-R., KD, TSC, LS: conducted research; CM-R, KD, TSC, LS: provided essential materials; GP: performed statistical analysis; GP, JCM, CM-R, CP, KD, TSC, LS, LR, EJS, OS, GMT, FFS, CWR, AA, AB, AMM, JW, TRH: wrote paper; GP: had primary responsibility for final content, and all authors: read and approved the final manuscript.

## Funding

The Newcastle 85+ Study was jointly funded by the Medical Research Council and Biotechnology and Biomedical Science Research Council (G0500997), now part of UK Research and Innovation (UKRI) in addition to the Newcastle Healthcare Charity. The following phases were funded by the Dunhill Medical Trust (R124/0509), Newcastle University, UK Medical Research Council and the British Heart Foundation (606013333). Overall, the Newcastle 85+ Study was supported by National Institute for Health Research Newcastle Biomedical Research Centre based at Newcastle upon Tyne Hospitals NHS Foundation Trust and Newcastle University. Analyses in the laboratory of LS were supported by Deutsche Forschungsgemeinschaft (DFG Research Unit 2558 TraceAge, Scho 849/6–2 and CRC/TR 296 “Local control of TH action,” LocoTact, P17). This work was part supported by the Medical Research Council (MRC) and Versus Arthritis as part of the Medical Research Council Versus Arthritis Centre for Integrated Research into Musculoskeletal Ageing (CIMA) [MR/R502182/1]. The MRC Versus Arthritis Centre for Integrated Research into Musculoskeletal Ageing is a collaboration between the Universities of Liverpool, Sheffield, and Newcastle. We would also like to acknowledge the generous donations provided by Cumbria Community Foundation and Beverley Charitable Trust Fund throughout the associated PhD program of study. The NuBrain consortium is funded by a UK Nutrition Research Partnership (UK NRP) Collaborative Award, an initiative supported by the Medical Research Council (MRC), Biotechnology and Biological Sciences Research Council (BBSRC), and the National Institute for Health Research (NIHR). The project reference is MR/T001852/1.

## Data availability

Data described in the manuscript are available upon request.

## Conflict of interest

LS holds shares on selenOmed GmbH, a company involved in selenium status assessment. All other authors report no conflicts of interest.
