# Supplementary material for: The association between selenium status and global and attention-specific cognition in very old adults in the Newcastle 85+ Study: cross-sectional and longitudinal analyses
Source: Am J Clin Nutr. 2024 Sep 11;120(5):1019–28. doi: 10.1016/j.ajcnut.2024.09.004 (PMC11600040; doi:10.1016/j.ajcnut.2024.09.004)
Supplement: multimedia component 1 [file mmc1.docx]

**Supplementary Material**

Participants with baseline selenium biomarkers and cognitive function measures

N = 755

1.5 years, N = 598

Attrition (20.8%)
Died 14.7 %; WD 3.6%, WD Health 2.5%

3 years N = 472

Attrition (21.1%)
Died 13.0%; Suspended 0.2%, WD 3.0%, WD Health 4.8%

Attrition (25%)

Died 20.1%, WD 2.3%, WD Health 2.5%

5 years, N = 354

Supplementary Figure 1: Flow chart of participants at each phase of the study and attrition percentage.

**Cognitive Assessments** with reference to Granic et al. 2015(56).

**CDR Attention Battery: Measures**

The following attention-specific measures/tasks were used as main outcomes:

1. **Simple Reaction Time (SRT)**: When the word “YES” was presented on the screen, the participants had to press the ‘YES’ response button as quickly as possible. Thirty ‘YES’ only stimuli were presented, with a varying inter-stimulus interval. Mean reaction times (standard deviation) were calculated in milliseconds (ms). Lower scores represented better performance.

2. **Choice Reaction Time (CRT)**: When the word ‘YES’ or ‘NO’ was presented on the screen, the participant had to press the corresponding button as quickly as possible. Thirty randomly chosen trials for each stimulus word was chosen with equal probability, with a varying inter-stimulus interval. Mean reaction times (standard deviation) were calculated in milliseconds (ms). Lower scores represented better performance.

3. **Digit Vigilance Task (DVT)**: At the right of the screen a target digit was randomly and constantly displayed. A series of digits (0-9) were presented in the centre of the screen at a rate of 150 per minute. When the digit in the series matched the target digit, the participant was required to press the ‘YES’ button as quickly as possible. There were three-hundred digits in the series, taking 2 minutes to complete. Mean reaction times (standard deviation) were calculated in milliseconds (ms). Lower scores represented better performance. Percentage (%) of accurate responses and false alarms (number of responses to non-targets) were also recorded.

4. **Power of Attention (PoA)**: A composite score was calculated by summing SRT, CRT and DVT mean reaction times (ms). This measures intensity of concentration: the faster the response, the more resources are being brought to complete the task. Lower scores represented better performance.

5. **Continuity of Attention (CoA)**: A composite score was calculated by combining the accuracy scores from the CDR and DVT (CRT accurate responses*0.30 + DVT accurate responses*0.30 –DVT false alarms). This measures the ability to sustain attention. Higher scores represented better performance.

6. **Reaction Time Variability (RTV)**: A composite score was calculated by summing up the coefficients of variance from SRT, CRT and DVT mean reaction times. This measures the fluctuation in attention and consistency in responding to correct target stimuli. Lower scores (coefficient of variance) represented better performance.

**Composite Score and Validation**

Principal component analysis (PCA) from a randomised, double-blinded, placebo-controlled trial (n=256, mean age 56±7 years) exploring the memory-enhancing effects of Ginkgo biloba and Panax ginseng in healthy individuals was used to empirically derive the weightings used for the CoA and PoA components(58).

**Supplementary Table 1** shows the baseline characteristics, for the cohort as a whole (n=755, mean age: 85.4±0.4 years, BMI: 24.4±4.3) and by tertiles of serum SELENOP concentration (< 2.32, 2.33-3.57 and ≥ 3.58 mg/L) and GPx3 activity (< 129.3, 120.3-166.1 and ≥166.1 U/L). Those with lower SELENOP were more likely be institutionalised (11.4 vs 4.0%, P = 0.004) compared to those with higher SELENOP concentrations. Females were more likely to have GPx3 activity in the highest tertile (65.2 vs 54.2%, P = 0.025).

**Attrition Descriptives**

Participants who were lost to follow-up at 3 years were more likely to be male (45.5% vs 37.5%), living in institution (22.8% vs 4.0%) and have higher disease counts (3 or more disease 51.6 % vs 35.3%). Although, education at 12+ years was lower (8.3% vs 14.5%), as was physical activity (44.6% vs 12.7%), selenium intakes (38.2 vs 39.6 µg/d) and status (serum selenium 48.0 vs 55.8 µg/L, SELENOP 2.8 vs 3.0 mg/L, GPx3 activity 128.6 vs 149.6 U/L). Similarly, participants who were lost to follow-up at 5 years were more likely to be male (46.5% vs 35.6%), living in institution (16.9% vs 4.0%) and have higher disease counts (3 or more disease 46.7% vs 34.9%). Education at 12+ years was lower (9.5% vs 15.1%), as was physical activity (36.4% vs 11.0%), selenium intakes (38.3 vs 39.8 µg/d) and status (serum selenium 50.7 vs 56.7 µg/L, SELENOP 2.9 vs 3.0 mg/L, GPx3 activity 134.3 vs 151.0 U/L) (data not shown). Participants who were lost to follow-up at 3 years were more likely to have higher cognitive impairment at baseline and 1.5 years, a higher prevalence of neurocognitive disease and higher scores for RTV and PoA and lower CoA, suggesting worse cognitive function. Likewise, participants who were lost to follow-up at 5 years were more likely to have greater cognitive impairment at 3 years, more neurocognitive disease, higher RTV and PoA scores, and lower CoA scores (**Supplementary Table 2**).

Supplementary Table 1: Baseline characteristics by serum selenoprotein P (SELENOP) and glutathione peroxidase 3 (GPx3) activity tertiles.

| Characteristic | All Participants | Selenium Status | | | | | | |  |  |
| --- | --- | --- | --- | --- | --- | --- | --- | --- | --- | --- |
|  |  | SELENOP mg/L | | | | GPx3 Activity U/L | | |  |  |
|  |  | < 2.32 | 2.33-3.57 | ≥ 3.58 | *P* | < 129.3 | 120.3-166.1 | ≥ 166.1 | |  |
| Participants, n | 755 | 254 | 250 | 251 |  | 251 | 252 | 250 | |  |
| Female, % (n) | 61.1 (461) | 59.4 (151) | 60.8 (152) | 62.9 (158) | 0.719 | 54.2 (136) | 63.5 (160) | 65.2 (163) | |  |
| Dietary selenium intake, µg/d | 45.3, 29.8 | 37.7, 26.1 | 38.6, 28.2 | 41.9, 32.8 | 0.054 | 37.4, 28.4 | 40.6, 26.1 | 40.6, 31.7 | |  |
| Serum selenium, µg/L | 54.9±19.0 | 44.2, 22.6 | 51.3, 18.1 | 66.0, 19.3 | <0.001 | 46.5, 23.6 | 52.1, 22.4 | 61.5, 20.7 | |  |
| SELENOP, mg/L | 3.1, 1.4 | 1.7, 0.8 | 2.9, 0.6 | 4.5, 1.3 | <0.001 | 1.9, 1.3 | 3.0, 1.5 | 4.0, 1.8 | |  |
| GPx3 Activity, U/L | 144.0±50.7 | 104.8±2.4 | 146.1±2.6 | 181.4±2.5 | <0.001 | 89.1±23.7 | 142.5±12.9 | 200.7±27.2 | |  |
| Waist:hip Ratio | 0.9 (0.1) | 0.9, 0.1 | 0.9, 0.1 | 0.9, 0.1 | 0.293 | 0.9, 0.1 | 0.9, 0.1 | 0.9, 0.1 | |  |
| Education | | | | | | | | |  |  |
| 0-9 years | 63.2 (477) | 58.2 (146) | 67.1 (165) | 67.5 (166) | 0.116 | 59.9 (148) | 69.5 (173) | 62.9 (154) | |  |
| 10-11 years | 23.2 (175) | 28.3 (71) | 19.9 (49) | 22.4 (55) |  | 27.1 (67) | 20.5 (51) | 23.3 (57) | |  |
| 12+ years | 12.1 (91) | 13.5 (34) | 13.0 (32) | 4.3 (25) |  | 13.0 (32) | 10.0 (25) | 13.9 (34) | |  |
| Occupation, %, n |  |  |  |  |  |  |  |  | |  |
| Managerial and Professional | 33.5 (253) | 36.3 (89) | 33.6 (79) | 35.3 (85) | 0.931 | 33.9 (82) | 38.2 (91) | 33.5 (80) | |  |
| Intermediate | 14.0 (106) | 15.5 (38) | 14.0 (33) | 14.5 (35) |  | 11.6 (28) | 14.7 (35) | 18.0 (43) | |  |
| Routine and Manual | 47.9 (362) | 48.2 (118) | 52.3 (123) | 50.2 (121) |  | 54.5 (132) | 47.1 (112) | 15.6 (116) | |  |
| Physical Activity, %, n |  |  |  |  |  |  |  |  | |  |
| Low | 21.5 (162) | 23.8 (60) | 24.3 (60) | 16.9 (42) | 0.167 | 23.0 (57) | 22.8 (57) | 19.4 (48) | |  |
| Medium | 42.6 (322) | 44.4 (112) | 40.9 (101) | 43.8 (109) |  | 42.7 (106) | 43.2 (108) | 43.5 (108) | |  |
| High | 35.0 (264) | 31.7 (80) | 34.8 (86) | 39.4 (98) |  | 34.3 (85) | 34.0 (85) | 37.1 (92) | |  |
| Number of Medications | 6.3, 3.8 | 6.0, 5.0 | 6.0, 4.0 | 6.0, 5.0 | 0.240 | 6.0, 5.0 | 6.0, 5.0 | 5.0, 5.0 | |  |
| Geriatric Depression Scale, %, n |  |  |  |  |  |  |  |  | |  |
| None | 73.9 (558) | 77.7 (181) | 79.8 (186) | 80.6 (191) | 0.938 | 79.2 (183) | 76.2 (179) | 82.6 (194) | |  |
| Mild | 11.7 (88) | 13.3 (31) | 12.0 (28) | 12.2 (29) |  | 12.1 (28) | 13.2 (31) | 12.3 (29) | |  |
| Severe | 7.5 (57) | 9.0 (21) | 8.2 (19) | 7.2 (17) |  | 8.7 (20) | 10.6 (25) | 5.1 (12) | |  |
| Institutionalised, % (n) | 8.9 (67) | 11.4 (29) | 11.2 (28) | 4.0 (10) | 0.004 | 10.8 (27) | 9.9 (25) | 6.0 (15) | |  |
| Alcohol drinkers, % (n) | 60.7 (458) | 61.3 (155) | 60.7 (150) | 61.0 (458) | 0.992 | 63.9 (159) | 58.6 (147) | 60.2 (150) | |  |
| Smokers, % (n) | 5.6 (42) | 6.7 (17) | 5.2 (13) | 4.8 (12) | 0.625 | 6.8 (17) | 4.8 (12) | 5.2 (13) | |  |
| Cardiovascular conditions, % (n) | 78.3 (591) | 75.5 (191) | 79.6 (199) | 80.1 (201) | 0.388 | 79.2 (198) | 77.0 (194) | 79.2 (198) | |  |
| Diabetes, % (n) | 14.2 (107) | 14.2 (36) | 11.6 (29) | 16.8 (42) | 0.250 | 14.7 (37) | 14.7 (37) | 13.2 (33) | |  |
| Dementia/Alzheimer/Parkinsons, % (n) | 9.1 (69) | 10.7 (27) | 8.8 (22) | 8.0 (20) | 0.559 | 7.6 (19) | 9.9 (25) | 10.0 (25) | |  |

Participants were compared between low, medium and high serum selenoprotein concentrations and GPx3 activity using chi-squared test for nominal values and Kruskal-Wallis for ordered and non-normally distributed data. All values represent median and IQR, unless otherwise stated. GPx3: glutathione peroxidase 3; hsCRP: high-sensitivity c-reactive protein; IL-6: interleukin 6; SELENOP: selenoprotein P; TNF-α:tumour-necrosis factor- alpha.

Supplementary Table 2: Cognitive function between participants and those who died during 3 and 5 year follow-ups.

| Characteristic | All Participants |  | | | | | |
| --- | --- | --- | --- | --- | --- | --- | --- |
|  |  | 3 years | | | 5 years | | |
|  |  | Participant | Died | *P* | Participant | Died | P |
| Baseline | |  | | | | | |
| SMMSE | 26.1±4.9 | 26.9±4.1 | 23.8±6.7 | <0.001 | 27.0±4.3 | 24.7±6.0 | <0.001 |
| PoA, ms | 1596.8±408.4 | 1484.6±196.7 | 1556.4±234.7 | 0.003 | 1475.5±196.9 | 1539.2±223.2 | 0.001 |
| CoA, ms | 51.8±8.7 | 53.1±7.5 | 47.8±11.4 | <0.001 | 53.4±7.6 | 49.3±10.4 | <0.001 |
| RTV, ms | 64.1± 19.5 | 62.0±23.9 | 69.4±23.9 | <0.001 | 61.2±15.6 | 67.9±24.2 | <0.001 |
| 1.5 years | |  | | | | | |
| SMMSE, n | 26.7±4.2 | 27.0±3.8 | 24.0±6.2 | <0.001 | 27.1±3.9 | 25.4±5.0 | <0.001 |
| PoA, ms | 1682.3±26.3 | 1575.3±310.5 | 1731.6±364.6 | <0.001 | 1569.2±308.2 | 1648.8±353.4 | 0.025 |
| CoA, ms | 51.8±0.4 | 52.5±7.4 | 47.5±10.8 | <0.001 | 53.0±7.1 | 49.5±9.8 | <0.001 |
| RTV, ms | 64.0±0.9 | 62.5±20.3 | 74.8±27.8 | <0.001 | 61.5±18.7 | 69.6±27.4 | <0.001 |
| 3 years | |  | | | | | |
| SMMSE, n | 25.5±5.3 |  |  |  | 26.1±4.7 | 23.3±6.9 | <0.001 |
| PoA, ms | 1690.3±31.9 |  |  |  | 1610.4±384.2 | 1713.6±451.4 | 0.087 |
| CoA, ms | 51.9±0.4 |  |  |  | 52.1±8.5 | 50.8±8.5 | 0.064 |
| RTV, ms | 63.1±1.1 |  |  |  | 62.1±21.8 | 66.0±19.7 | 0.013 |

CoA: continuity of attention; GPx3: glutathione peroxidase activity; PoA: power of attention; RTV: reaction time variability; SMMSE: standardized mini mental state examination.

**Sensitivity Analyses**

At baseline, participants in the highest tertile of serum selenium concentration had SMMSE that was on average .58 points (SE=0.27, P=0.032) greater than those in the lowest tertile after adjustment for all covariates (**Supplementary Table 3**). This association was maintained over 5-year follow-up; those participants in the highest tertile of serum selenium concentration had a lower rate of decline in SMMSE compared with those in the lowest tertile in the fully adjusted model (β =0.04 (0.02) P=0.022) (**Supplementary Table 4**).

Supplementary Table 3: Relationships between measures of cognition and tertiles of each biomarker of selenium status (serum selenium, glutathione peroxidase 3 activity, selenoprotein P)*.*

| Outcome | Serum Selenium | | Selenoprotein P | | GPx3 Activity | |
| --- | --- | --- | --- | --- | --- | --- |
|  | β (SE) | *p* | β (SE) | *p* | β (SE) | *p* |
| SMMSE Model 1 Middle | 1.42 (0.43) | 0.001 | -0.06 (0.44) | 0.896 | -0.42 (0.44) | 0.348 |
| Highest | 2.27 (0.43) | <0.001 | 0.59 (0.44) | 0.184 | -0.08 (0.44) | 0.857 |
| Model 2 Middle | 0.33 (0.26) | 0.208 | -1.00 (0.26) | 0.713 | -0.32 (0.26) | 0.229 |
| Highest | 0.58 (0.27) | 0.032 | 0.22 (0.26) | 0.411 | -0.38 (0.27) | 0.153 |
| PoA Model 1 Middle | -14.80 (20.60) | 0.473 | -6.46 (20.57) | 0.750 | 17.80 (20.50) | 0.385 |
| Highest | -34.30 (20.50) | 0.094 | -4.92 (20.17) | 0.810 | 39.20 (20.20) | 0.053 |
| Model 2 Middle | 0.28 (20.04) | 0.989 | -5.34 (19.82) | 0.788 | -0.09 (19.88) | 0.996 |
| Highest | -13.24 (20.34) | 0.515 | -2.15 (19.69) | 0.913 | 32.02 (19.76) | 0.106 |
| CoA Model 1 Middle | 1.39 (0.76) | 0.068 | -0.32 (0.76) | 0.679 | 1.31 (0.76) | 0.088 |
| Highest | 2.54 (0.76) | <0.001 | 0.70 (0.75) | 0.355 | 0.74 (0.76) | 0.328 |
| Model 2 Middle | -0.04 (0.68) | 0.950 | -0.60 (0.67) | 0.372 | 0.88 (0.68) | 0.196 |
| Highest | 0.60 (0.69) | 0.391 | -0.41 (0.67) | 0.546 | 0.05 (0.68) | 0.943 |
| RTV Model 1 Middle | -4.16 (1.81) | 0.022 | -1.56 (1.81) | 0.390 | 0.15 (1.82) | 0.935 |
| Highest | -5.38 (1.80) | <0.01 | -2.91 (1.79) | 0.105 | -0.35 (1.80) | 0.844 |
| Model 2 Middle | -2.81 (1.77) | 0.114 | -2.49 (1.75) | 0.156 | 0.55 (1.76) | 0.757 |
| Highest | -3.39 (1.81) | 0.061 | -2.80 (1.75) | 0.111 | 0.75 (1.77) | 0.671 |

CoA: continuity of attention; GPx3: glutathione peroxidase activity; PoA: power of attention; RTV: reaction time variability; SE: standard error SMMSE: standardized mini mental state examination. Model 1: adjusted for biomarker of interest; Model 2: adjusted for biomarker of interest, sex, physical activity, waist:hip ratio; education, geriatric depression score (GDS), disease count, presence of all diabetes, cardiovascular and neurocognitive conditions, smoking status, alcohol and selenium intake. The lowest selenium biomarker was set as the comparator. Lower β scores for PoA, RTV and higher β scores for CoA and SMMSE indicate better function. Se, SePP = 621 GPx3 = 619 PoA, RTV, Co, SMMSE = 623, 621, 639 637.

Supplementary Table 4: Associations between tertiles of selenium status biomarkers at baseline and cognitive decline (SMMSE) over 5 years and composite scores of cognition over 3 years using generalized linear mixed models.

| Outcome | Serum Selenium | | Selenoprotein P | | GPx3 Activity | |
| --- | --- | --- | --- | --- | --- | --- |
|  | β (SE) | *p* | β (SE) | *p* | β (SE) | *p* |
| SMMSE^1^ Model 1 Middle | 0.05 (0.02) | 0.021 | -1.07^E-3^ (0.02) | 0.962 | -0.01 (0.03) | 0.570 |
| Highest | 0.09 (002) | 0.002 | 0.03 (0.02) | 0.126 | 0.01 (0.02) | 0.720 |
| Model 2 Middle | 0.03 (0.02) | 0.087 | -0.01 (0.02) | 0.653 | -0.02 (0.02) | 0.394 |
| Highest | 0.04 (0.02) | 0.022 | 0.02 (0.02) | 0.368 | -0.01 (0.02) | 0.768 |
| PoA^2^ Model 1 Middle | -0.01 (0.02) | 0.620 | 0.02 (0.02) | 0.194 | 0.05 (0.02) | 0.026 |
| Highest | -0.03 (0.02) | 0.110 | 0.02 (0.02) | 0.284 | 0.04 (0.02) | 0.044 |
| Model 2 Middle | -2.86^E-3^ (0.02) | 0.889 | -0.01 (0.02) | 0.798 | 0.02 (0.02) | 0.145 |
| Highest | -0.02 (0.02) | 0.316 | -3.82^E-3^ (0.02) | 0.850 | 0.02 (0.02) | 0.193 |
| CoA^2^ Model 1 Middle | 0.06 (0.03) | 0.026 | -0.01 (0.03) | 0.732 | 3.72^E-3^ (5.58^E-3^) | <0.001 |
| Highest | 0.09 (0.03) | 0.001 | 0.03 (0.03) | 0.343 | -1.75^E-3^ (5.40^E-3^) | 0.001 |
| Model 2 Middle | 1.66^E-3^ (0.02) | 0.942 | -0.03 (0.02) | 0.214 | 0.01 (0.02) | 0.660 |
| Highest | 0.01 (0.02) | 0.538 | -0.01 (0.02) | 0.515 | -0.01 (0.02) | 0.525 |
| RTV^2^ Model 1 Middle | -0.06 (0.03) | 0.060 | -0.01 (0.03) | 0.800 | 5.91^E-3^ (0.03) | 0.850 |
| Highest | -0.09 (0.03) | 0.005 | -0.04 (0.03) | 0.230 | -2.96^E-3^ (0.03) | 0.920 |
| Model 2 Middle | -0.03 (0.03) | 0.318 | -0.02 (0.03) | 0.408 | 0.02 (0.03) | 0.618 |
| Highest | -0.05 (0.03) | 0.088 | -0.04 (0.03) | 0.239 | 0.01 (0.03) | 0.728 |

CoA: continuity of attention; GPx3: glutathione peroxidase activity; PoA: power of attention; RTV: reaction time variability; SE: standard error SMMSE: standardized mini mental state examination. Time: change over time; ^1^ decline over 5 years; ^2^ decline over 3 years. Results produced from generalized linear mixed models. Model 1: adjusted for biomarker of interest and time; Model 2: adjusted for biomarker of interest, time and their interaction, sex, physical activity, waist:hip ration; education, geriatric depression score, disease count, presence of all diabetes, cardiovascular and neurocognitive conditions, smoking status, alcohol intake and selenium intake. For all models, tertile 1, the lowest concentration, was used as the reference (0.00). Lower β scores for PoA, RTV and higher β scores for CoA and SMMSE indicate better function. SMMSE N = 753, 588, 441, 313 for baseline, 1.5, 3 and 5 year follow up; CoA and PoA N = 705, 528, 395 and RTV N = 702, 528, 393 for baseline, 1.5 and 3 year follow up.

**Sensitivity Analyses**

At baseline, after removing participants with neurocognitive conditions (Parkinsons and Alzheimer’s disease) and adjusting for institutionalization the significant association between tertiles of serum selenium and SMMSE was lost (β 0.48 (0.26) P=0.069). There was a significant association between tertiles of GPx3 activity and SMMSE in the fully adjusted model (β -0.52 (0.26) P=0.046), but not with the continuous values of GPx3 activity (β -1.98^E-3^ (2.08^E-3^) P=0.343). In the prospective models, the significant association between tertiles of serum selenium and SMMSE remained (β 0.04 (0.02) P=0.035), although the effect size was small. This association was not evident when using continuous values of serum selenium (β 0.01 (0.01) P=0.484) (data not shown).


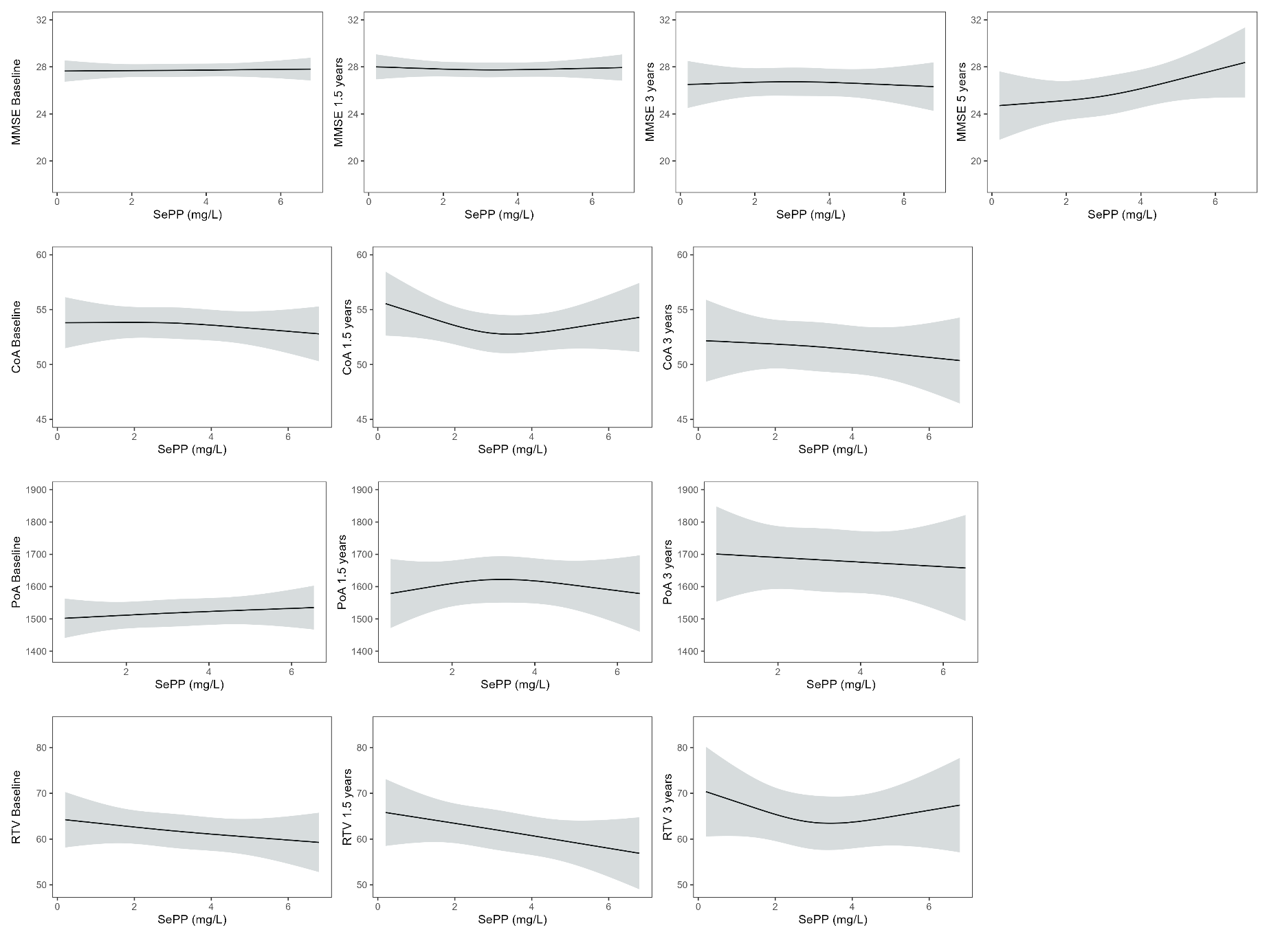


Supplementary Figure 2: Restricted cubic splines for selenoprotein P and: standardized mini mental state examination (MMSE), continuity of attention (CoA), power of attention (PoA) and reaction time variability (RTV), using a priori knots at 5, 10 and 90th percentiles. Estimates from the fully adjusted models are depicted by solid lines and 95% confidence intervals are depicted by the shaded areas. Models are adjusted for biomarker of interest, sex, physical activity, waist:hip ratio; education, geriatric depression score (GDS), disease count, presence of all diabetes, cardiovascular and neurocognitive conditions, smoking status and alcohol intake and selenium intake.


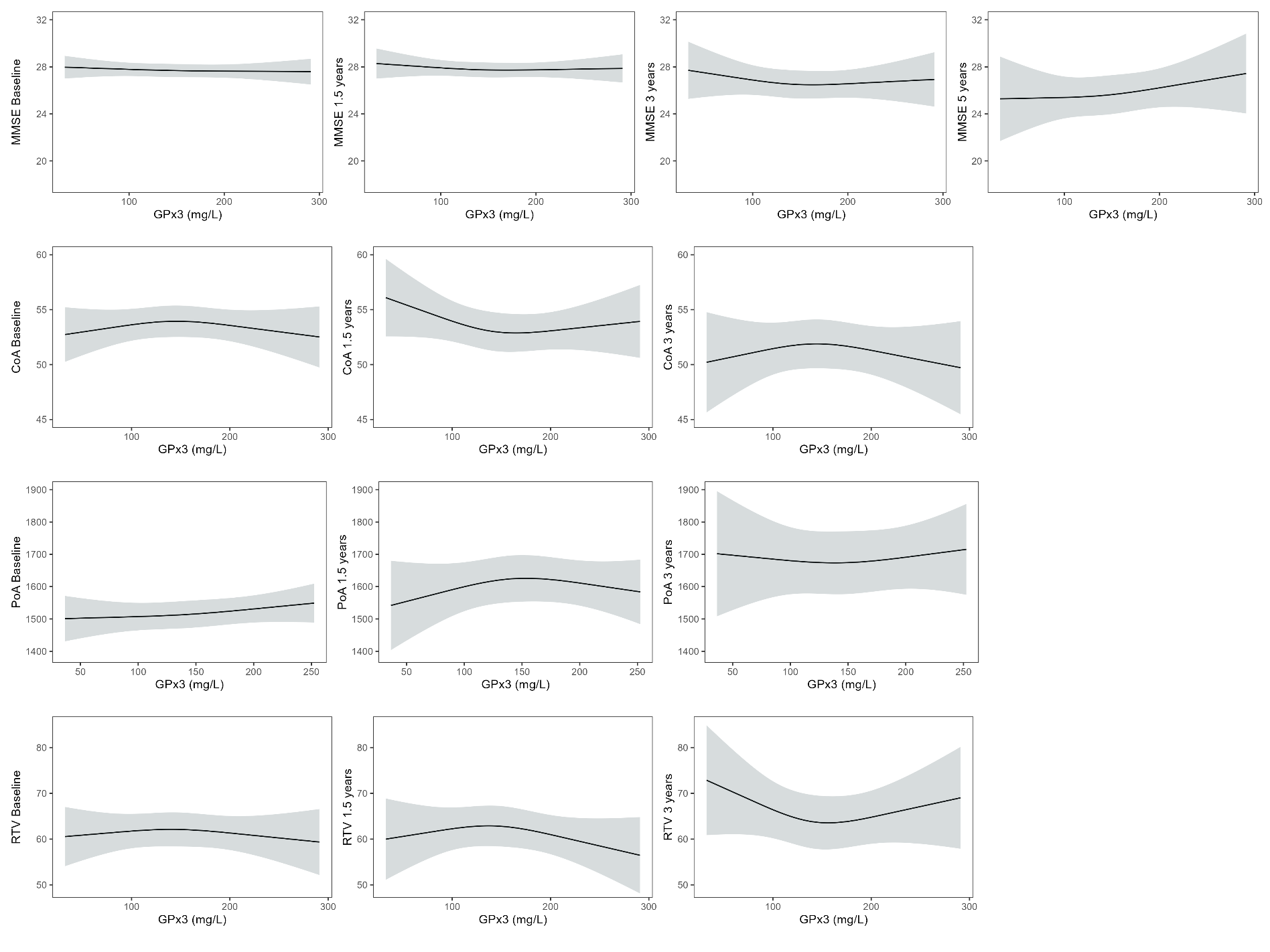


Supplementary Figure 3: Restricted cubic splines for glutathione peroxidase 3 activity (U/L) and: standardized mini mental state examination (MMSE), continuity of attention (CoA), power of attention (PoA) and reaction time variability (RTV), using a priori knots at 5, 10 and 90th percentiles. Estimates from the fully adjusted models are depicted by solid lines and 95% confidence intervals are depicted by the shaded areas. Models are adjusted for biomarker of interest, sex, physical activity, waist:hip ratio; education, geriatric depression score (GDS), disease count, presence of all diabetes, cardiovascular and neurocognitive conditions, smoking status and alcohol intake and selenium intake.
